# Examining decentralization and managerial decision making for child immunization program performance in India

**DOI:** 10.1016/j.socscimed.2022.115457

**Published:** 2023-01

**Authors:** Isabelle Feldhaus, Susmita Chatterjee, Emma Clarke-Deelder, Logan Brenzel, Stephen Resch, Thomas J. Bossert

**Affiliations:** a Department of Global Health and Population, Harvard T.H. Chan School of Public Health, Boston, MA, USA; b The George Institute for Global Health, New Delhi, India; c Swiss Tropical & Public Health Institute, Allschil, Switzerland; d Bill & Melinda Gates Foundation, Seattle, WA, USA; e Center for Health Decision Science, Harvard T.H. Chan School of Public Health, Boston, MA, USA

**Keywords:** Decentralization, Decision space, Immunization, Health services delivery, Program performance, Structural equation modeling, Factor analysis, India

## Abstract

Despite widespread adoption of decentralization reforms, the impact of decentralization on health system attributes, such as access to health services, responsiveness to population health needs, and effectiveness in affecting health outcomes, remains unclear. This study examines how decision space, institutional capacities, and accountability mechanisms of the Intensified Mission Indradhanush (IMI) in India relate to measurable performance of the immunization program.

Data on decision space and its related dimensions of institutional capacity and accountability were collected by conducting structured interviews with managers based in 24 districts, 61 blocks, and 279 subcenters. Two measures by which to assess performance were selected: (1) proportion reduction in the DTP3 coverage gap (i.e., effectiveness), and (2) total IMI doses delivered per incremental USD spent on program implementation (i.e., efficiency). Descriptive statistics on decision space, institutional capacity, and accountability for IMI managers were generated. Structural equation models (SEM) were specified to detect any potential associations between decision space dimensions and performance measures.

The majority of districts and blocks indicated low levels of decision space. Institutional capacity and accountability were similar across areas. Increases in decision space were associated with less progress towards closing the immunization coverage gap in the IMI context. Initiatives to support health workers and managers based on their specific contextual challenges could further improve outcomes of the program.

Similar to previous studies, results revealed strong associations between each of the three decentralization dimensions. Health systems should consider the impact that management structures have on the efficiency and effectiveness of health services delivery. Future research could provide greater evidence for directionality of direct and indirect effects, interaction effects, and/or mediators of relationships.

## Highlights

• Study found key relationships between decision space, capacity, and accountability.
• SEM can be used to detect linkages between decentralization and performance.
• Increased managerial decision space was associated with increased capacity.
• Increased decision space was associated with less progress closing coverage gaps.
• Study may support centralization of immunization services for hard-to-reach groups.

## 1 Introduction

Driven by a convergence of political and economic factors in the late 1980s, major international development agencies advocated for decentralization reforms in health sectors as a means to improve the responsiveness, efficiency, and quality of services in low- and middle-income countries (Collins and Green, 1994). Published literature acknowledges that the effectiveness of decentralization to achieve health system objectives depends on an adequate endowment of financial resources, institutional capacities to manage changing processes, and genuine accountability of local authorities to the interests of their constituents (Ahmad and Brosio, 2009; Bardhan and Mookherjee, 2006; Bossert et al., 2015; Cobos Muñoz et al., 2017; Mitchell and Bossert, 2010; Saito, 2006). Dwicaksono and Fox (2018) proposed a pathway linking decentralization with health system performance (Dwicaksono and Fox, 2018). In their framework, decentralization policy dictates the decision space of decision makers in the health system (Bossert, 1998). Bossert defines ‘decision space’ as the degree of choice that local authorities exercise across the functional areas of the health system (Bossert, 1998). These choices determine inputs into the health system that result in health system performance (Dwicaksono and Fox, 2018).

**Fig. 1:**
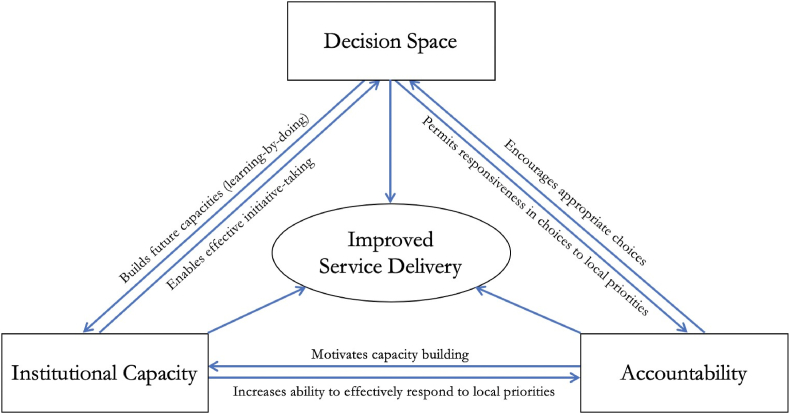
Conceptual framework of synergies between decentralization and service delivery. Source: Bossert and Mitchell (2011).

Studies consistently point to the necessity of adequate capacity and accountability to effectively exercise decision space (Alonso-Garbayo et al., 2017; Bossert and Mitchell, 2011; Heywood and Choi, 2010; Maharani and Tampubolon, 2014). Institutional capacities can enable effective decision-making while increasing officials’ ability to effectively respond to local priorities (Fig. 1). For example, if budgeting decisions are decentralized to the district level, then it is critical that the district have sufficient capacity to budget, such as staff trained in finance or budgeting. Similarly, accountability can motivate capacity building and encourage appropriate priority setting and decision making: if local managers are empowered to make hiring decisions, then they should be held accountable for hiring the appropriate human resources (Bossert and Mitchell, 2011). Few articles explicitly address the interdependent relationships between decision space, institutional capacities, and accountability or detail the nature of these relationships (Eslie Roman et al., 2017; Liwanag and Wyss, 2019; McCollum et al., 2018). Innovations in research approaches can further this area and our understanding of how to improve the delivery of health services.

### 1.1 Impacts of decentralization on childhood immunization

Studies of decentralization's impact on childhood immunization highlight key features of the decision space framework and their critical roles in health services delivery. In his study examining the impact of political decentralization on childhood immunization across 138 countries, Khaleghian found that decentralized middle-income countries have lower coverage rates than centralized middle-income countries (Khaleghian, 2004). One explanation offered for these findings was that, without sufficient central direction to make immunization a health priority, local authorities may face a greater diversity of demands from the local community, driving negative impacts on immunization coverage. Another explanation suggested that, because health services, including immunization, are among the most visible manifestations of government presence at the local level, these services may be a key focus of public demands in low-income countries where fewer public services are offered.

Maharani and Tampubolon showed that fiscal decentralization reforms in Indonesia failed to improve the efficiency, quality, and equity of child immunization service delivery. When increasing local discretion over funds also failed to improve outcomes, the wide variation in how budgets were used led authors to conclude that local capacity to manage budgets according to local needs was paramount in achieving reform objectives (Maharani and Tampubolon, 2014).

### 1.2 Study objective

This study conducts an in-depth examination of the decision space, institutional capacities, and accountability mechanisms functioning within the Intensified Mission Indradhanush (IMI), a program of periodic intensification of routine immunization implemented by the Government of India in low-coverage districts across India, and assesses how these features relate to performance of the immunization program. We extend current approaches to researching how decision space and, more broadly, decentralization affects health services delivery. Based on survey data from IMI officials across managerial levels, we quantitatively describe decision space, capacity, and accountability features across levels of the program as latent constructs driving service delivery. Quantitative estimations of these constructs are then analyzed with respect to the outcomes of the IMI program using a structural equation modeling approach.

## 2 Materials and methods

### 2.1 Organizational structure of the health system and child immunization in India

India has 29 states and a total population of 1.28 billion (66% rural and 34% urban) (Central Intelligence Agency, n.d.). At the national level, the Ministry of Health and Family Welfare (MoHFW) establishes policies and guidelines for the implementation of country-wide programs. The states oversee the implementation and supervision of these various programs and provide infrastructure and curative services. The district links state structures and peripheral structures, such as community health centers, primary health centers, and subcenters within blocks, the administrative units below districts. Subcenters provide primary health care services, including vaccinations, to populations between 3000 and 5,000, while higher levels of care (i.e., block, district) provide specialized health care services.

The districts are responsible for working and coordinating with their states for the implementation and supervision of immunization. State and district task forces are responsible for monitoring the Universal Immunization Program (UIP), launched in 1985. The extent to which decision space, institutional capacity, and accountability varied across levels of the program was unclear, prompting examination of these features across district, block (i.e., sub-district), and subcenter (i.e., primary health care facility) levels of implementation.

UIP is tasked with reaching a birth cohort of ∼26 million infants and ∼30 million pregnant women every year. The MoHFW funds the national immunization program, providing technical assistance and policy guidance to states. UIP provides vaccines to protect against diphtheria, pertussis, childhood tuberculosis, poliomyelitis, pneumococcal pneumonia, measles, neonatal tetanus, hepatitis B, *Haemophilus* influenza B, rotavirus, and Japanese Encephalitis (in endemic areas). Having been operational for over 30 years, UIP has fully immunized 65% of children in the first year of life and has continued efforts to further increase immunization nationwide (International Institute for Population Sciences (IIPS) and ICF, 2017).

In 2017–2018, the Government of India implemented IMI, focusing on low-coverage districts across India (Gurnani et al., 2018). This program is an example of periodic intensification of routine immunization (PIRI) (World Health Organization, 2009). The aim of the program was to cover all children missed by the UIP and earlier initiatives in select districts and urban cities to achieve 90% coverage by December 2018. These locations were recorded to have low immunization coverage at the time of IMI implementation.

Program management was decentralized to district and block levels (Gurnani et al., 2018). Within each selected district or city, a process of microplanning was carried out to identify children with missing doses and outreach to communities to deliver immunization at temporary vaccination sites during one week of each month for four consecutive months. Microplanning involved stakeholders at the district (i.e., District Immunization Officer, DIO), block (i.e., Medical Officer In-Charge, MOIC), and subcenter (i.e., Auxiliary Nurse Midwives, ANMs) levels. Based on the IMI Operational Guidelines, MOICs in coordination with DIOs were responsible for planning IMI sessions, including sites for vaccination, availability of human resources, and special strategies for reaching underserved, resistant, and/or reluctant communities. ANMs were deployed to identified areas to deliver vaccine doses. Task forces for immunization at the state, district, and block levels were formed for regular planning and review of IMI. Monitoring data was intended to be fed back to block, district, and state task forces for immediate corrective action and to guide programmatic decision making.

Given these role functions and processes, there is the possibility for variation in decision space, institutional capacity, and accountability across health system levels and locations. For instance, DIOs in Uttar Pradesh may have greater purview over how many ANMs they allocate to deliver IMI sessions compared to those in Rajasthan, who may be required to gain approvals from higher levels regarding resource allocations. Decision space for IMI could encompass not only human resources management, but also development of microplans, organization of service delivery, and supervisory actions. Institutional capacity could include measures of educational qualifications, vaccination-related trainings completed, level of experience, funding received, and infrastructure of facilities where IMI sessions were held. Accountability mechanisms could be indicated by how often supervisors communicate, check registers, provide feedback, or conduct site visits.

### 2.2 Data collection

To assess decision space and its related dimensions, key informant interviews were conducted in a sample of IMI districts between July 2018 and January 2019. Five states with high concentration of IMI activity (Assam, Bihar, Maharashtra, Rajasthan, and Uttar Pradesh) were selected for inclusion. Within these states, 34 districts and six urban areas were selected. In Uttar Pradesh and Maharashtra, urban areas were selected at random. In Bihar and Rajasthan, IMI was conducted in one urban district, both of which were included in this study. No urban districts in Assam were selected for IMI. To select rural districts, districts were grouped into divisions, which were selected at random using probability proportional-to-size (PPS) sampling. Within districts, 30% of blocks were selected at random. Within blocks, 10% of subcenters were selected at random. A total of 90 blocks and 289 subcenters were selected.

Questionnaires were developed specific to district, block, and subcenter levels. Data collectors were trained in best practices of qualitative interviewing, study objectives, and each question to ensure data quality and consistency. The DIO, MOIC, and ANM at the district, block, and subcenter levels, respectively, were interviewed. Questionnaires were adapted from past survey tools aimed at assessing decision space, institutional capacities, and accountability in India (Bossert and Mitchell, 2011; World Bank Group, 2010).

Questionnaires were pilot tested in the first three districts visited for data collection and revised for clarity and appropriateness to context. Information on respondents’ age, sex, and highest level of education was collected. The instrument measured resources (e.g., availability of funds) as well as processes (e.g., programmatic training, supportive supervision, responsiveness to feedback, monitoring and evaluation activities). Data collectors gathered information on the quantity of IMI activities (i.e., training and microplanning meetings, number of IMI sessions, and number of supervision visits) and resources (i.e., staffing of IMI sessions). Interviews were supplemented by IMI forms, microplans, and ANM records. Data from the Demographic Health Survey (DHS) 2015-16 was used to collate indicators for basic vaccination coverage at the district level.

Data was collected using paper forms, from which data was transferred into electronic format within 24 h of interviews. Data was stored using Kobo Toolbox, a secure cloud-based data storage tool.

### 2.3 Data analysis

Two measures by which to assess performance with respect to program effectiveness and efficiency, respectively, were selected: (1) proportion reduction in the DTP3 coverage gap, and (2) total IMI doses delivered per incremental dollar (USD) spent on IMI implementation (not including vaccine costs).

(1) $Effectiveness=\frac{n_{doses}}{\left(1-coverage_{DTP3}\right)*n_{births}}$
(2) $Efficiency=\frac{n_{doses}}{program\;cost}$

These indicators intend to represent the degree to which IMI closed the coverage gap in program areas. DTP3 coverage, a standard measure of immunization coverage, indicates that children have been reached for all three doses of the DTP vaccine in the first year of life. The proportion reduction in the DTP3 coverage gap is a relative measure. If a district had 90% coverage and improved to achieve 95%, it would have reduced its gap by 50%. Moving from 80% to 90% or from 98% to 99% would also indicate a gap reduction of 50%. This approach accounted for potential ceiling effects, recognizing that it may be more challenging to increase coverage as coverage approaches 100%.

District coverage and birth cohort estimates were computed and outlined in related work as part of a larger study of IMI (Clarke-Deelder et al., 2021). The unit of coverage is the proportion of vaccinated children in a district. To determine the coverage gap, coverage estimates were subtracted from one. To determine the number of unvaccinated children in a district, the coverage gap was multiplied by the 2017 surviving birth cohort size in the district. Surviving birth cohort size was computed using data on the child population size as well as birth and neonatal mortality rates in 2016 and 2017 (Clarke-Deelder et al., 2021; International Institute for Population Sciences (IIPS) and ICF, 2017; World Bank, 2017). Those children receiving a vaccine dose during the date range of IMI were assumed to have been vaccinated under the program.

Because only respondents who were working during the implementation of IMI could speak to their roles, responsibilities, and experiences during IMI activities, only data from respondents present during IMI implementation were eligible for analysis.

All data analysis was conducted in R (version 3.6.1).

### 2.4 Quantification of decision space, institutional capacity, and accountability

Data was coded into dummy variables representing key program indicators falling within the pre-defined domains of the decision space framework (Table 1) (Bossert, 1998; Bossert and Mitchell, 2011). Indicators within each domain (e.g. strategic and operational planning, human resources management, service delivery and organization, and governance for the dimension of decision space) were summed for each observation. Summing the indicators within each domain resulted in a ‘domain score’ for each of the domains within each dimension (i.e., decision space, institutional capacity, and accountability). Within each level (i.e., separately for districts, blocks, and subcenters), domain scores were scaled between 0 and 2 for ease of interpretation and comparison within the sample and on the scale of past published literature (Bossert and Mitchell, 2011).

**Table 1:** Indicators comprising each framework dimension and domain.

DECISION SPACE	INSTITUTIONAL CAPACITY	ACCOUNTABILITY
**Strategic and operational planning**	**Education**	**Supervisor presence**
Development of microplan	Highest educational qualifications obtained	Presence of direct supervisor
Partner engagement		Frequency of communication
Supervision of lower levels	**Experience**	
Staff training	Years in government service	**Supervisor actions**
Participation in review meetings	Years in present location	Feedback
Joint/consensus-based decision making		Checked logistics
Changes implemented during IMI	**Management training**	Checked supplies
*Microplan*	Number of trainings attended	Checked adherence to microplan
*Session site*	Ability to solve problems (self-assessed)	Conducted site visit
Community engagement	Sufficient training for role	House to house verification
**Human resources management**		Observed service delivery
Re-allocated human resources	**Funding**	Motivated families
Staff training	Allocated funds	Checked registers^a^
Motivation of staff	Received sufficient funds	
Changes implemented during IMI		**Community participation**
*Staff training*	**Adoption of technology**	Influence of community
**Service organization and delivery**	Implementation of electronic registers	
Coordination role		
Motivation of staff	**Infrastructure**^a^	
Changes implemented during IMI	Electricity	
*Session site*	Flush or pour toilet	
Changes made to RI	Pit latrine	
*Session site*	Useable toilet facilities	
*Number of sessions held*	Running water	
*Day of week of session*	Separate room for ANC	
*Inclusion of households/coverage*^a^	Standalone building	
**Governance and local participation**	Government/private building	
Supervisory actions	Owned/rented building	
*Checked logistics*		
*Checked supplies*		
*Checked adherence to microplan*		
*Conducted site visit*		
*House to house verification*		
*Observed service delivery*		
*Motivated families*		
Made changes based on feedback		
Community participation		

a Used for subcenter level only.

For example, for the strategic and operational planning domain of the decision space dimension, if a DIO reported being involved in (1) development of the IMI microplan, (2) engaging partners, (3) supervising lower levels, (4) training staff, and (5) participating in review meetings, his district would have an unscaled strategic and operational planning domain score of 5. Suppose that when all of the scores for the strategic and operational planning domain have been determined across districts, the resulting range of scores is between 0 and 7. Each individual score would then be rescaled to lie between 0 and 2 to allow for comparability across domains and dimensions. Thus, an unscaled score of 5 would correspond to a scaled score of 1.4 and an unscaled score of 2 would correspond to a scaled score of 0.6. An overall domain score of 0 in ‘strategic and operational planning’ represents low or narrow decision space for decisions within this domain; a score of 2 represents high or wide decision space.

The respective domain scores of each dimension were averaged to result in final composite scores for (i) decision space, (ii) institutional capacity, and (iii) accountability. Average scores around 1, the midpoint between 0 and 2, could indicate that, on average, scores for that dimension at that level are moderate. Average scores below or above 1 would suggest a central tendency towards higher or lower values for that dimension at that level of the system.

Bolded text indicates domains within dimensions of decision space, institutional capacity, and accountability.

‘Institutional capacity’ was defined according to Bossert and Mitchell: the ability of individuals or systems to perform appropriate functions across administrative, technical, and organizational dimensions (Bossert and Mitchell, 2011). Questionnaires collected information on work experience, training, funding, adoption of technology, and infrastructure available for IMI sessions. ‘Accountability’ referred to the structures in place to ensure that decisions made by non-elected officials (i.e., health managers) responded to local needs (Bossert and Mitchell, 2011). Measures of accountability considered the presence of a supervisor, that supervisor's specific actions over lower levels, and how communities participated in how program decisions were made. Together, these scores provide a quantitative heuristic indication of decentralization that can be compared across and within states, districts, blocks, and subcenters.

### 2.5 Structural equation model: measurement model, specification, and estimation

Structural equation modeling (SEM) combines factor analysis and regression analysis to analyze structural relationships between measured variables and latent constructs (Bollen and Noble, 2011). SEM is a theory-driven approach, requiring a theoretical framework that form the basis of model specification (Kline, 2005). In the decision space framework, the dimensions of decision space, institutional capacities, and accountability mechanisms can be considered latent constructs, measured by a set of observed indicators. The framework and literature applying the conceptualization of decision space provide a strong basis that prompts further exploration in this direction. For these reasons, SEM was selected as a quantitative technique to explore how decentralization dimensions relate to observed performance metrics. At this stage, the findings of this analysis do not presume to act as evidence for causation, but rather point towards additional tools to detect key relationships.

Confirmatory factor analysis (CFA), the first step of this approach, uses a hypothesized model to estimate a covariance matrix representing survey respondents, which is then compared to what has been observed (Schreiber et al., 2006). Each latent variable was defined by the corresponding set of discrete observed indicators collected in the survey data to reflect each framework dimension (Cai, 2012). Sets of the indicators from the survey data that fall within the decision space, institutional capacity, and accountability dimensions in the literature were tested to identify measurement models that made theoretical sense, were reasonably parsimonious, and acceptably corresponded to the data (Kline, 2005). The variance-covariance matrix of model parameters was used to estimate the model.

SEM techniques require large sample sizes to support the robustness of findings. Due to concerns related to sample size, only block and subcenter levels were included for analysis using SEM. For simplicity, models were assumed to be recursive, meaning that error residuals are uncorrelated and any effects are unidirectional without feedback loops (Kline, 2005). Using the *lavaan* R package, maximum likelihood estimation was used to estimate the model (Kline, 2005). Model fit was assessed using the Tucker-Lewis index (TLI), comparative fit index (CFI), and root mean square error of approximation (RMSEA), reported as relevant criteria for categorical data (Schreiber et al., 2006). TLI and CFI values greater than 0.90, and RMSEA values below 0.08 were considered an acceptable fit for measurement models (Schreiber et al., 2006).

## 3 Results

### 3.1 Profile of respondents

A total of 24 districts, 61 blocks, and 279 subcenters were included in the analysis (Table 2). District-level managers tended to be older than at lower levels. All respondents at the district and block levels completed at least graduate level education. Respondents working at higher levels tended to have more years of experience in government service. At the district and block levels, men held the DIO and MOIC roles, while all of the ANMs were female. Most often, respondents’ roles related to microplan development. Higher levels were involved in supervisory and coordination activities as well as partner (i.e., departmental stakeholders or international agencies) engagement. ANMs interacted with communities more often. Nearly all DIOs participated in review meetings during IMI implementation. Fewer MOICs were involved in these meetings. A majority reported that decisions made during review meetings with higher managerial levels were made jointly and/or by consensus. Nearly all, respondents indicated having a direct supervisor and communicating with that supervisor nearly every day.

**Table 2:** Summary of survey responses by level.

Indicator	District	Block	Subcenter
	N = 24	N = 61	N = 279
**State**			
Assam	4 (16.7%)	3 (4.9%)	28 (10.0%)
Bihar	3 (12.5%)	9 (14.8%)	44 (15.8%)
Maharashtra	4 (16.7%)	11 (18.0%)	60 (21.5%)
Rajasthan	3 (12.5%)	10 (16.4%)	30 (10.8%)
Uttar Pradesh	10 (41.7%)	28 (45.9%)	117 (41.9%)
**Age (mean, years)**	51.5	45.2	41.8
**Sex**			
Male	21 (87.5%)	55 (90.2%)	0 (0.0%)
Female	3 (12.5%)	6 (9.8%)	279 (100.0%)
**Education**			
Secondary school	0 (0.0%)	0 (0.0%)	123 (69.5%)
Graduate	15 (79.0%)	53 (86.9%)	68 (24.5%)
Post-Graduate	4 (21.1%)	8 (13.1%)	16 (5.8%)
**Years in government service, mean**	22.4	16.2	15.0
**Years in present location, mean**	11.75	6.1	7.8
**Training received to implement IMI**			
IMI microplanning and reporting workshop	17 (77.3%)	49 (84.5%)	7 (2.7%)
IMI communication session	9 (40.9%)	14 (24.1%)	0 (0.0%)
AEFI protocol training	5 (22.7%)	6 (10.3%)	0 (0.0%)
BRIDGE IPC skills training	3 (13.6%)	7 (12.1%)	16 (6.2%)
Media spokesperson training	3 (13.6%)	2 (3.5%)	0 (0.0%)
CSO orientation	1 (4.6%)	2 (3.5%)	0 (0.0%)
Health workers training	1 (4.6%)	3 (5.2%)	241 (93.8%)
Mobilizers training	1 (4.6%)	2 (3.5%)	16 (6.2%)
**Sufficient funds allocated to district**			
Yes	14 (58.3%)	38 (63.3%)	–
No	5 (20.8%)	15 (25.0%)	–
Don't know	5 (20.8%)	7 (11.7%)	–
**Role in IMI planning**			
Microplan development	16 (66.7%)	40 (65.6%)	213 (76.9%)
Training	7 (29.2%)	16 (26.2%)	0 (0.0%)
Supervision	4 (16.7%)	10 (16.4%)	0 (0.0%)
Coordination	11 (45.8%)	22 (36.1%)	0 (0.0%)
Partner engagement	13 (54.2%)	2 (3.3%)	0 (0.0%)
Community engagement	5 (20.8%)	13 (21.3%)	110 (39.7%)
**Participated in IMI review meetings**	23 (95.8%)	45 (76.3%)	–
**Decision making process**			
Joint decision	10 (43.5%)	19 (42.2%)	–
Others made final decision	9 (39.1%)	12 (26.7%)	–
**Supervisor actions received**			
Reviews microplan	10 (41.7%)	29 (51.8%)	169 (62.1%)
Provides feedback	12 (50.0%)	18 (32.1%)	2 (0.74%)
Conducts site visit	12 (50.0%)	27 (48.2%)	51 (18.8%)
Checks supplies	–	13 (23.2%)	133 (48.0%)
Conducts house verification	–	6 (10.7%)	57 (21.0%)
Motivates families	–	3 (5.4%)	95 (34.9%)
Motivates staff	–	7 (12.5%)	–
Checks logistics	–	–	47 (17.3%)
Observes service delivery	–	–	42 (15.4%)
Checks register	–	–	38 (14.0%)
**Frequency of supervisor communication**			
Almost daily	13 (58.3%)	40 (71.4%)	257 (93.1%)
2–4 times per week	5 (20.8%)	14 (25.0%)	6 (2.2%)
About once a week	2 (8.3%)	0 (0.0%)	4 (1.5%)
Less than 1 time per month	3 (12.5%)	0 (0.0%)	3 (1.1%)
**Reported community participation**	7 (29.2%)	8 (13.1%)	51 (18.4%)
**Reported changes to RI**			
Sites added	13 (56.5%)	19 (43.2%)	13 (9.7%)
Sessions added	2 (8.7%)	9 (20.5%)	35 (26.2%)
Additional households included in RI	2 (8.7%)	13 (29.6%)	69 (51.5%)
Improved follow up	6 (26.1%)	2 (4.5%)	3 (2.2%)
**Adoption of electronic RCH registers**			
Fully implemented	13 (54.2%)	45 (73.8%)	–
Began implementation	9 (37.5%)	11 (18.0%)	–
Have not started	1 (4.8%)	4 (7.6%)	–

Of the total 40 districts, 91 blocks, and 281 subcenters where data were collected, 24 districts (60%), 61 blocks (67%), and 279 subcenters (97%) were eligible for inclusion. Reasons for exclusion included: (i) being new in the DIO, MOIC, or ANM position (i.e., hired after IMI implementation), (ii) unavailability, absence, or refusal for interview, or (iii) not being involved in IMI implementation. The reduced proportion of respondents at the district and block levels reporting having been involved in IMI suggests that there was turnover or migration in roles and/or stations at these levels. Fewer changes in human resources appeared at lower levels. At subcenters, some interviews could not be conducted because the ANM was too busy providing services during the time of the research team's visit.

Of those districts eligible for analysis, 2% of data was missing with two observations missing responses to as many as four questions. Observations at the block level were missing 7% of data, while 9% of data was missing for subcenter observations. At the subcenter level, 2% of the data for analysis was missing.

### 3.2 Summary of health services delivery and decision space

Based on DHS data from 2016, DTP3 coverage in the participating rural districts ranged from 16.3% to 83.3%, with a mean of 65.3% and a median of 66.9% (International Institute for Population Sciences (IIPS) and ICF, 2017). Data on costs per dose delivered for UIP have been previously reported by Chatterjee et al. (2018): the weighted average national level cost per child vaccinated with the third dose of the DPT vaccine was US$31.67 (2017 USD) with costs ranging between US$20.08 and US$34.81 (Chatterjee et al., 2018). For the sample of districts included in this study, doses delivered per thousand USD ranged from 0.37 to 98 with a mean of 18 and median of 10 doses.

Across district, block, and subcenter levels, the mean scores for institutional capacity and accountability were close to 1, indicating similar levels of institutional capacity and accountability at the three levels (Table 3). Results suggest that there were a handful of districts and blocks with significantly higher decision space than others, but that the majority indicated lower decision space. Within the decision space dimension, human resources management was shown to have the lowest score. The decision space scores were, on average, higher at the subcenter level than the district or block level. This difference was driven by greater strategic and operational planning and governance and local participation scores at the subcenter level.

**Table 3:** Mean composite scores for dimensions and domains by level.

Dimension/Domain	District	Block	Subcenter
	N = 24	N = 61	N = 279
	μ(SD)	μ(SD)	μ(SD)
**Decision Space**	**0.70 (0.38)**	**0.67 (0.32)**	**0.97 (0.32)**
Strategic and operational planning	0.89 (0.52)	0.85 (0.55)	1.16 (0.64)
Human resources management	0.37 (0.50)	0.37 (0.37)	–
Service delivery and organization	0.65 (0.52)	0.60 (0.52)	0.42 (0.54)
Governance and local participation	0.90 (0.56)	0.84 (0.52)	1.34 (0.38)
**Institutional Capacity**	**1.06 (0.40)**	**1.08 (0.40)**	**0.99 (0.32)**
Education	1.21 (0.42)	1.13 (0.34)	1.05 (0.75)
Experience in government service	0.96 (0.52)	0.59 (0.71)	0.58 (0.77)
Duration in present location	1.07 (0.78)	0.50 (0.61)	0.70 (0.75)
Additional training	0.92 (0.49)	1.63 (0.54)	1.46 (0.53)
Funding	1.48 (0.67)	1.45 (0.74)	–
Adoption of technology	1.52 (0.59)	1.68 (0.60)	–
Infrastructure^a^	–	–	1.28 (0.59)
**Accountability**	**1.06 (0.63)**	**0.89 (0.38)**	**1.06 (0.35)**
Supervision presence	1.56 (0.68)	1.73 (0.56)	1.91 (0.37)
Supervisory actions	1.42 (0.65)	0.74 (0.44)	0.93 (0.52)
Community participation	0.58 (0.93)	0.26 (0.68)	0.37 (0.78)

a Rural subcenters only.

Institutional capacity as measured by experience in government service and length of time stationed in the respondents’ present location was lower for blocks and subcenters compared to other indicators of capacity. The majority of subcenters reported high levels of supervision as measured by having a direct supervisor and frequency of communication, while community participation to the extent that it influenced IMI operations and implementation strategy remained relatively low across levels.

**Fig. 2:**
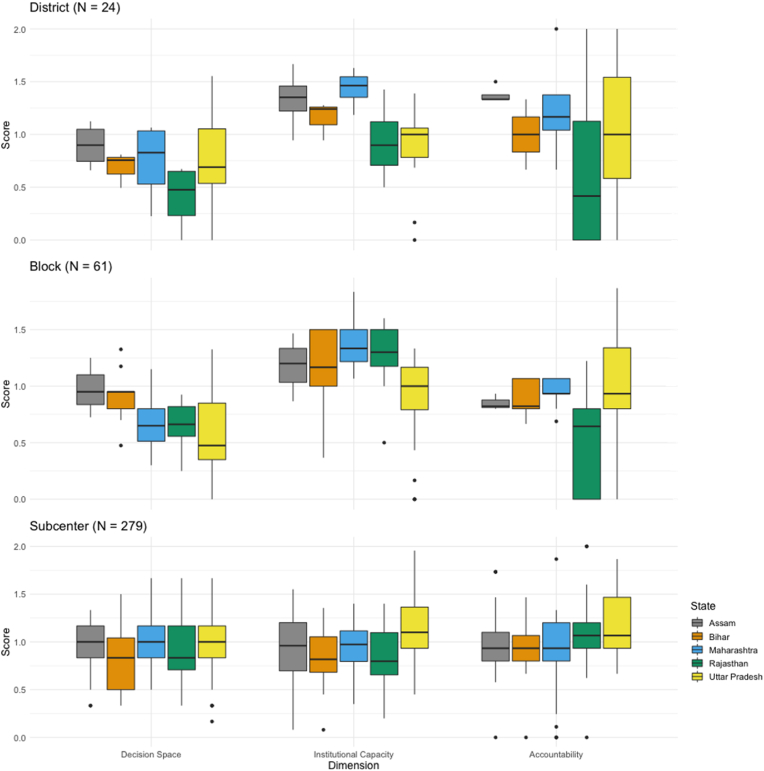
Decentralization scores by dimension and state.

Comparison of dimensions measured across levels revealed variation by state, with the largest variation at the district level (Fig. 2). At the district level, managers located in Rajasthan consistently reported lower scores compared to other states. Given that scores at the block and subcenter levels are not excessively high, this could suggest that Rajasthan engages in more centralized decision making at the state level compared to other states. Respondents in Maharashtra consistently reported higher scores, as did managers in Assam. However, the district-level sample size was small for these states. Block-level analysis showed that managers in Rajasthan reported lower levels of accountability, but block-level respondents in Uttar Pradesh reported the lowest decision space and institutional capacity. Results showed that subcenters reported the least variation across states.

Defining framework dimensions and their associations with program performance.

Specification of the model was guided by Bossert's decision space framework (Fig. 1) (Bossert, 1998; Bossert and Mitchell, 2011). Decision space was defined by having a joint decision-making process in place and the relevance of community participation. Institutional capacity was defined by the time an ANM and MOIC had spent working in a location, suggesting that experience in the area and familiarity with its context was particularly relevant. Accountability was defined by actions taken by supervisors to oversee IMI activities, specifically whether MOICs regularly received feedback from their supervisor and whether MOICs were involved in mobilizing vaccine-hesitant households to support ANMs in their block.

**Fig. 3:**
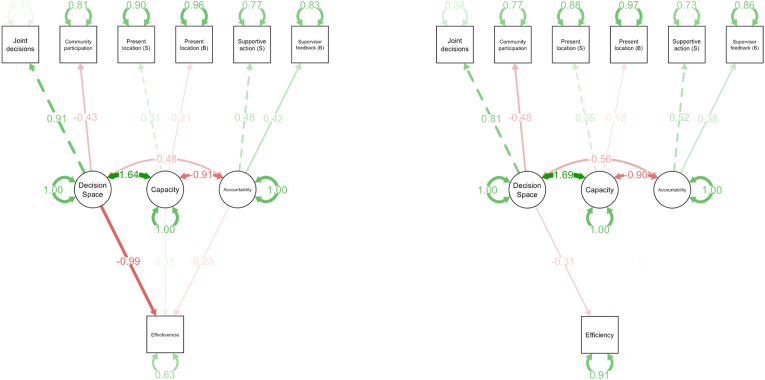
Path diagram for structural equation models of program effectiveness and efficiency with standardized estimatesVariances and covariances between variables are indicated by arrows and noted values. Red arrows indicate negative variances. Green arrows indicate positive variances. B = block-level indicators; S = subcenter-level indicators. (For interpretation of the references to colour in this figure legend, the reader is referred to the Web version of this article.)

Model results illustrate strong interrelationships between decision space, capacity, and accountability at the block and sub-center levels (Fig. 3). Increased decision space as measured by having a joint decision-making process and community participation was associated with lower reductions in the DTP3 coverage gap (Table 4, Table 5). Institutional capacity and accountability constructs were not significantly associated with program effectiveness or efficiency measures. Controlling for age, sex, and education of local managers slightly altered estimates of associations of decision space with program efficiency, highlighting the inverse relationship as statistically significant (Table 5).

**Table 4:** Estimates of relationships between decision space variables and program effectiveness.

	Unadjusted				Adjusted			
	Unstandardized			Standardized	Unstandardized			Standardized
	Estimate (SE)	z	*p*	Estimate (SE)	Estimate (SE)	z	*p*	Estimate (SE)
Factor Loadings								
*Decision Space*								
Joint decision-making process	1.00^a^			0.45 (0.91)	1.00^a^			0.47 (0.95)
Community participation	−0.32 (0.08)	−4.20	0.00	−0.20 (−0.58)	−0.30 (0.06)	−5.37	0.00	−0.14 (−0.41)
*Institutional Capacity*								
Time in current location (MOIC)	1.00^a^			1.55 (0.31)	1.00^a^			1.69 (0.34)
Time in current location (ANM)	−1.07 (0.31)	−3.48	0.00	−1.65 (−0.21)	−0.90 (0.22)	−4.19	0.00	−1.52 (−0.19)
*Accountability*								
Supervisor feedback (MOIC)	1.00^a^			0.22 (0.48)	1.00^a^			0.23 (0.48)
Supervisor took supportive action (ANM)	0.90	0.42	0.03	0.20 (0.42)	0.88 (0.29)	3.07	0.00	0.20 (0.41)
Regression Slopes								
*Effectiveness: Proportion reduction in DTP3 coverage gap*								
Decision Space	−0.87 (0.23)	−3.71	0.00	−0.39 (−0.99)	−0.70 (0.15)	−4.76	0.00	−0.33 (−0.86)
Institutional Capacity	0.04 (0.06)	0.76	0.45	0.07 (0.17)	0.03 (0.04)	0.75	0.45	0.05 (0.12)
Accountability	−0.41 (0.45)	−0.92	0.36	−0.09 (−0.23)	−0.45 (0.30)	−1.50	0.13	−0.10 (−0.26)
ANM								
*Age*	–				0.07 (0.02)	4.18	0.07	0.07 (0.21)
*Education*	–				−0.01 (0.04)	−0.23	0.82	−0.01 (−0.01)
MOIC								
*Age*	–				−0.05 (0.02)	−2.53	0.01	−0.05 (−0.12)
*Sex: Female*	–				0.10 (0.07)	1.38	0.17	0.10 (0.07)
*Education*	–				0.20 (0.06)	3.07	0.00	0.20 (0.15)
Fit Indices								
AIC	2764.23				7063.65			
CFI	0.96				0.51			
TLI	0.92				0.29			
RMSEA	0.06				0.17			

a Fixed parameter.

**Table 5:** Estimates of relationships between decision space variables and program efficiency.

	Unadjusted				Adjusted			
	Unstandardized			Standardized	Unstandardized			Standardized
	Estimate (SE)	z	*p*	Estimate (SE)	Estimate (SE)	z	*p*	Estimate (SE)
Factor Loadings								
*Decision Space*								
Joint decision-making process	1.00^a^			0.40 (0.81)	1.00^a^			0.40 (0.81)
Community participation	−0.41 (0.09)	−4.45	0.00	−0.16 (−0.48)	−0.42 (0.07)	−6.13	0.01	−0.17 (−0.49)
*Institutional Capacity*								
Time in current location (MOIC)	1.00^a^			1.74 (0.35)	1.00^a^			1.77 (0.35)
Time in current location (ANM)	−0.85 (0.29)	−2.93	0.00	−1.47 (−0.18)	−0.82 (0.21)	−3.88	0.00	−1.45 (−0.18)
*Accountability*								
Supervisor feedback (MOIC)	1.00^a^			0.25 (0.52)	1.00^a^			0.24 (0.52)
Supervisor took supportive action (ANM)	0.75 (0.37)	2.04	0.04	0.18 (0.38)	0.76 (0.27)	2.79	0.01	0.19 (0.38)
Regression Slopes								
*Efficiency: Doses delivered per US$1K spent on IMI*								
Decision Space	−15.41 (9.50)	−1.62	0.11	−6.22 (−0.31)	−17.73 (7.13)	−2.49	0.01	−7.09 (−0.35)
Institutional Capacity	−0.01 (1.23)	−0.01	0.99	−0.02 (0.00)	0.01 (0.92)	0.02	0.99	0.03 (0.00)
Accountability	−1.70 (15.50)	−0.11	0.91	−0.42 (−0.02)	−0.92 (11.74)	−0.08	0.94	−0.23 (−0.01)
ANM								
*Age*	–				0.67 (1.06)	0.64	0.52	0.67 (0.04)
*Education*	–				−3.30 (2.62)	−1.26	0.21	−3.30 (−0.07)
MOIC								
*Age*	–				1.41 (1.20)	1.18	0.24	1.41 (0.07)
*Sex: Female*	–				1.41 (1.20)	0.95	0.34	4.41 (0.06)
*Education*	–				−1.12 (4.01)	−0.28	0.78	1.12 (0.02)
Fit Indices								
AIC	4015.77				9368.46			
CFI	0.98				0.38			
TLI	0.95				0.11			
RMSEA	0.04				0.17			

a Fixed parameter.

## 4 Discussion

### 4.1 Summary of key findings

This analysis extends current research approaches to decision space, institutional capacity, and accountability by first applying approaches seen in the literature to quantify each of these dimensions for IMI in India and then, applies an SEM approach to analyze to what extent they can be quantitatively associated with program performance. Similar to other studies of decision space, there was variability in decision space, institutional capacity, and accountability across states. CFA at block and subcenter levels revealed that not all indicators loaded onto decision space, capacity, and accountability. Parsimonious measurement models resulted in the best goodness-of-fit measures; each latent factor in the final model was generated based on two items.

This is a first example of how SEM can be applied to detect linkages between decentralization and performance. The relationships shown do not point to causality, however. We were constrained by our limited knowledge of the mechanisms at play as well as temporality of the phenomena in question. Rather, this model revealed decision space to be associated with the effectiveness of IMI towards closing India's immunization coverage gap.

That more decision space at the block and subcenter levels was associated with lower performance could be explained in the event that those managers able to decide whether to conduct sessions actually conduct fewer sessions based on the information available to them. In some areas, the lack of a subcenter building or other infrastructure limited staff in their ability to implement IMI. In other areas, safety from violence was a concern among staff. It is also possible that those with higher levels of decision space implemented fewer IMI sessions given their other responsibilities. The model reveals an inverse association between greater community participation and decision space: delegating influence to citizens could result in less decision space in the hands of IMI managers. Households may have played a greater role in managers’ decisions to not pursue vaccination among unreached hesitant households. The direction of influences that decrease decision space, whether from higher levels that encourage greater vaccination or lower levels, where the most outspoken may be either champion or hesitant households, may drive the nature of decision space impacts on performance. The role of community participation in both limiting decision space and improving performance could be further explored.

Examination of the interdependent relationships between dimensions revealed both similarities and differences with previous studies. Similar to the findings of Bossert and Mitchell, model results showed strong associations between decision space and capacity and capacity and accountability, while accountability and decision space were positively, though not as strongly, associated with each other (Bossert and Mitchell, 2011). In our study, as decision space increased, capacity increased. A possible explanation for this is that greater institutional capacity offers more opportunity to exercise decision space or vice versa. Alongside this relationship, capacity and accountability covaried inversely to a lesser degree – diverging from the findings of Bossert and Mitchell. Similarly, as decision space increased, accountability decreased. While these relationships may be surprising given the emphasis on a positive association between the dimensions in the Bossert & Mitchell framework, a possible explanation is that, with increasing decision space and/or capacity, fewer formal accountability structures may be in place or deemed necessary. Fewer formal accountability structures in place may drive wider decision space among managers, for example, or indications of greater capacity may compel the institution of fewer formal accountability mechanisms.

The CFA that drove the definition of the latent dimensions in this model resulted in a different, narrower definition of institutional capacity than was posited in Bossert's and Mitchell's study in Pakistan (Bossert and Mitchell, 2011). Bossert and Mitchell defined capacity by both resources (e.g., availability of funds, adequacy of infrastructure/staff) and processes (e.g., monitoring and evaluation activities) (Bossert and Mitchell, 2011). Our study defined capacity by the time a health worker had spent in a particular location. It may be that new individuals were brought into challenging areas or to replace health officials failing to reach coverage goals. As capacity is currently measured in our model, such an occurrence would result in low indications of capacity despite wide decision space and high accountability processes in place. High levels of accountability would further support such a narrative as detection of poor performance of previous human resources allocated to the area could prompt reassignment of health officials. These results demonstrate the nuance required in interpretation of findings from these types of models as well as highlight how these models can capture organizational behaviors and relationships.

Our results contribute to the evidence that these three features of decentralization remain intimately related. However, it is notable that decision space and accountability were also more narrowly defined due to our measurement approach. Rather than drawing from each of the functional domains outlined in the framework, only indicators from strategic and operational planning and governance and local participation domains were included based on the CFA. This may suggest that, in the context of IMI, decision making surrounding human resources management and service organization and delivery domains were not correlated with items relevant to decision space and accountability. This may align with our understanding of managers operating at these levels, where human resources management and service organization decisions are likely primarily determined at higher managerial levels.

Similarly, the CFA results defined accountability differently than other studies. Bossert and Mitchell defined accountability by asking elected officials how much initiative they took in health sector decision-making processes and asking civil servants whether they were consulted or interacted with local representatives on health sector issues (Bossert and Mitchell, 2011). Receiving feedback and support on how to handle context-specific situations, rather than actions taken to oversee operations (e.g., verifying adherence to protocols, checking supplies, and observing service delivery), comprised the accountability construct in this study. This definition focuses more on the actions of a supervisor rather than the involvement of local bodies or communities. In the context of IMI, therefore, supervisory actions may have a larger influence in accountability than community actions. This also aligns with our understanding of hard-to-reach populations, which includes households hesitant to receive vaccines or government-delivered health services and whose views on health services may contradict those of traditional health systems.

### 4.2 Implications of findings for immunization programs and health systems

IMI was introduced to improve immunization coverage alongside existing efforts to deliver child vaccines, including UIP and broader primary health initiatives, such as Village Health and Nutrition Days (VHNDs). Immunization is one of the core activities of VHNDs, held once a month to deliver primary health care services to underserved areas. However, it remains unclear how IMI implementation may affect such initiatives (Johri et al., 2019). Understanding decision space, capacity, and accountability structures in ways that allow for improved performance of IMI could also have positive implications for the operations of other primary health care initiatives serving underserved areas.

Based on the findings of this analysis, investments that drive support of vaccination at grassroots levels – such as training and identifying champions within communities, for example – may reduce decisions space of blocks and subcenters, but have positive impacts on the effectiveness of immunization programs (Dadari et al., 2021; Stamidis et al., 2019). Building appropriate accountability mechanisms may also have the desired impacts on decision space and program performance. More effective accountability mechanisms in the Indian immunization context may manifest as greater supportive supervision rather than solely oversight of immunization activities. Supportive supervision could include greater involvement of higher levels in motivating hesitant households, identifying more tractable solutions for areas prone to violence and demonstrations, and advocating for sufficient resources for areas without existing infrastructure for health services delivery. Future iterations of IMI or existing immunization programs in India could place greater emphasis on these context-specific management to tackle child immunization objectives in these challenging, diverse areas.

Research to-date largely agrees that relationships or synergies exist between decision space, institutional capacity, and accountability across different contexts (Bossert and Mitchell, 2011; Eslie Roman et al., 2017). The findings of this study support earlier findings of the important interrelationships among these dimensions. Considering how decentralization of management influences processes to achieve immunization coverage and the delivery of other critical health services can provide key insights into the functioning of the broader health system. Study findings, by highlighting the role of lower level management, emphasizes additional opportunities for improvement of immunization programs through enabling effective decision making to reach more people with essential health services. Future research could provide greater evidence for directionality of direct and indirect effects, interaction effects, and/or mediators of relationships.

Considering how the health system and its programs empower their staff to make decisions may be critical in promoting the effectiveness of health service delivery and improving adequate access to essential services. It may be important to purposefully and strategically allocate decision space to ensure achievement of appropriate program goals. In the IMI context, increases in decision space were associated with fewer doses delivered towards addressing the immunization coverage gap. This finding addresses one of the persistent questions in the decentralization and immunization literature raised by Khaleghian and others and may support arguments for the centralization of immunization activities, even in situations of already limited local decision space. In cases in which managers made decisions based on local information, it is also possible that initiatives to support health workers based on their specific contextual challenges could further improve outcomes of the program.

### 4.3 Limitations

Limitations of this analysis include the sample size of the data studied. SEMs require large sample sizes to generate consistent results. This motivated use of lower level health services delivery outcomes. As a result, the model reveals less about the aggregate performance outcomes at the district level. Uttar Pradesh comprises a majority of the sample analyzed, and as a result, phenomena in this region may be driving some of the findings presented here disproportionately more than other states. Tests for validity and reliability demonstrated that only the decision space construct showed sufficient convergent validity across the models examined. Future research would benefit from a larger sample size as well as a focus on improving the composite reliability, convergent validity, and divergent validity measures across the framework dimensions.

SEMs in this paper were assumed to be recursive and models were simplified to increase the likelihood of model convergence. Nevertheless, it is possible that a non-recursive, more complex model would more appropriately convey the relationships occurring in decentralized health systems. This was a first iteration towards understanding how such a health system could be best represented using a structural model and may continue to be built upon in future research.

Since data were collected using key informant interviews with open-ended questions and retrospective review of records, this study is subject to the limitations imposed by these methods of data collection. Responses gathered via interview may be influenced by the interviewer herself. Tool development processes and researcher training aimed to minimize the potential effect of bias as a result of interviewing or questionnaire design. It is possible that respondents’ answers may have been influenced by their superiors should it have been impossible to conduct interviews in private settings. Questions that were believed to be unduly biased by such a presence were removed from the analysis for this reason.

The fact that only data from respondents present during the implementation of IMI could be used for analysis may have contributed to selection bias. Random staffing changes would not have contributed to bias. However, it may be that less successful managers vacated their positions or more successful managers moved into different positions in short periods of time; three district-level managers and four block-level managers had not been involved in IMI at the time interviews were conducted, pointing to staff changes between IMI implementation and the time of this study. In districts and blocks, 60% and 67% of data was eligible for inclusion, respectively. In a hypothetical scenario in which data for less successful managers who would have reported lower decision space and institutional capacity were missing, our study may have overestimated the negative association between these dimensions and performance measures. Similarly, if most of the data missing were due to the movement of successful managers who would have reported low levels of decision space and capacity to other positions, analyses would have underestimated the magnitude of these relationships.

This study differs from other studies of decision space and its related dimensions due to the arguably more limited scope of the IMI program. For example, other studies of decision space, institutional capacity, and accountability highlight the availability of funds as a key item informing these dimensions. In the case of IMI, the resources and processes behind how availability of funds was allocated was considered uniform within managerial levels. The lack of variability of this variable and others made it irrelevant to include as a measure, and may contribute to differences in findings and/or incompatibility with other studies of decision space. Furthermore, our data-driven (as opposed to theory-driven) measurement approach in the CFA resulted in narrowly-defined measures of the constructs of interest (decision space, institutional capacity, and accountability) which, while reflective of the covariance structures present in our data, may not fully align with our theoretical understandings of these constructs.

Our study analyzed data on the vaccine doses delivered through the IMI program. It is possible that some of the children who were vaccinated through IMI would have been vaccinated even in the absence of IMI; this would make it appear that IMI performance was better than it was in practice.

As is often a limitation in the evaluation of immunization programs, it remains challenging to measure improvements in immunization coverage without a reliable denominator representing the total number of children that need immunization services. Proxy measures of program effectiveness and efficiency were used to assess IMI performance.

### 4.4 Future research

Future research should aim to build on this work and address its limitations. In scenarios of increased decision space, it may be critical to identify motivations and frameworks for health workers’ decision making in order to predict its effects. Larger sample sizes or pooled datasets may provide improved opportunities to examine features of decentralization and health system performance using SEM approaches. Considering how approaches could be applied using data from routinely administered or widely used surveys may be a way to address this challenge. Given the highly contextual nature of decentralization, it remains challenging to achieve high external validity in this area. Yet, systematic approaches to analysis can yield a consistent framework by which to assess and understand research findings.

## 5 Conclusions

Study findings emphasize the potential role of decision space to implement health delivery systems efficiently and effectively for improved child immunization. Health systems should consider the impact that management structures have on the efficiency and effectiveness of health services delivery. Increases in decision space were associated with less progress towards closing the immunization coverage gap in the IMI context. Initiatives to support health workers based on their specific contextual challenges could further improve outcomes of the program. With the possibility for greater insights and understanding into the relationships between decision space, capacity, and accountability for service delivery, research should continue to explore the potential of innovative approaches to inform strategic planning and implementation of health policies and programs.

## Ethical clearance

Ethical approval for this study was received from the […].

## Credit author statement

**Isabelle Feldhaus**: Conceptualization, Methodology, Formal analysis, Writing – original draft, Writing – review & editing, Visualization; **Susmita Chatterjee**: Investigation, Data curation, Supervision, Project administration; **Emma Clarke-Deelder**: Formal analysis, Writing – review & editing; **Logan Brenzel**: Supervision, Writing – review & editing; **Stephen Resch**: Conceptualization, Supervision, Funding acquisition; **Thomas J. Bossert**: Conceptualization, Methodology, Supervision.

## Data Availability

Data will be made available on request.
