# Supplementary material for: Examining decentralization and managerial decision making for child immunization program performance in India
Source: Soc Sci Med. 2023 Jan;317:115457. doi: 10.1016/j.socscimed.2022.115457 (PMC9870749; doi:10.1016/j.socscimed.2022.115457)
Supplement: Multimedia component 1 [file mmc1.docx]

**Appendix**

**A Study instruments**

Study instruments were developed based on past questionnaires used in studies examining decision space in India (World Bank Group, 2010). They were further adapted to context and the IMI program based on discussions throughout the training of the locally-based research team and pilot testing of the tools. These instruments made up the final section of a larger questionnaire collecting data on IMI from retrospective review of facility records and key informant interviews.

| 1 |  |  |
| --- | --- | --- |
| 2 |  |  |
| 3 |  |  |

code

**Section 5 of 5: Decision capacity**

State: Data collector:

District: Date:

*Respondent profile General Overview*

| D5.01 | Age (years) : |
| --- | --- |
| D5.02 | Sex : ▢ Male ▢ Female |
| D5.03 | Years in government service : |
| D5.04 | Length of time (*years*) in present district : |
| D5.05 | Current position :  ▢ DIO ▢ Other, specify: |
| D5.06 | Were you involved in IMI in any role?  ▢ YES ▢ NO**, END INTERVIEW** |
| D5.07 | Position during IMI :  ▢ DIO ▢ Other, specify: |
| D5.08 | Were you based in this district during IMI?  ▢ YES ▢ NO, specify location: |
| D5.09 | Educational qualifications (list highest degree) |
| D5.10 | Have you received any additional formal management training related to your work in the past 2 years  Duration (specify #  No Title of training days/weeks/months) |

|  | I am going to ask you some questions about the Routine Immunization program (RI) in your district. |
| --- | --- |
| D5.11 | In your district, has there been progress toward immunization coverage goals in the past 2 years? |
| D5.12 | What are the main challenges of implementing RI in your district?  Encourage free response; note free responses here or use tick boxes if applicable:  ▢ Staffing / Vacancies ▢ Funding  ▢ Inefficiencient operations ▢Vaccine Supply Issues  ▢ Patients lack awareness ▢ Patients hesitancy  ▢ Target population hard to reach ▢ Social media rumors  ▢ Staff motivation ▢ Record-keeping issues |
| D5.13 | How effective has IMI been as a supplement to RI?  ▢ NOT AT ALL EFFECTIVE  ▢ SOMEWHAT EFFECTIVE  ▢ EFFECTIVE  ▢ VERY EFFECTIVE |

code

*Strategic and operational planning*

| D5.16 | Did you participate in **IMI review meetings** during IMI rounds?  ▢ YES ▢ NO, skip to D5.19 |
| --- | --- |
| D5.17 | What is the process for making decisions during IMI review meetings? |
| D5.18 | What changes were made to IMI implementation following the IMI review meetings, if any? |

| D5.14 | What are the **top three** challenges that you have experienced implementing IMI? (List in order of importance)  1.  2.  3. |
| --- | --- |
| D5.15 | What is your role / what specific actions do you take in the development of the final plan of IMI? |

code

*Financial Management*

| D5.24 | In what way was the training not sufficient? What additional training would have helped you? |
| --- | --- |
| D5.25 | Has your experience with the implementation of IMI led you to make any changes to the RI plan or the way RI is implemented?  ▢ YES ▢ NO, skip to D5.27 |
| D5.26 | If yes, how? |
| D5.27 | Do you think there will be a need for additional rounds of IMI in your district in the future? Why or why not? |

| D5.19 | Were funds allocated to your district specifically for IMI?  ▢ YES ▢ NO |
| --- | --- |
| D5.20 | Did your district have sufficient funds to implement IMI as planned?  ▢ YES ▢ NO |

*Human resources*

| D5.21 | Describe the training that you received to implement IMI. (check all that apply)  ▢ IMI communication session  ▢ BRIDGE IPC Skills training  ▢ AEFI protocol training  ▢ CSO orientation on communication plan and use of monitoring tools  ▢ Media spokespersons training  ▢ IMI microplanning & reporting workshop (district-level)  ▢ Health workers training (ANMs, LHVs, health supervisors)  ▢ Mobilizers training (ASHAs, AWWs)  ▢ Other, specify: |
| --- | --- |
| D5.22 | Did you use this training to solve any problems with IMI planning or implementation in the last year?  ▢ YES ▢ NO |
| D5.23 | Do you feel that this training was sufficient given your responsibilities under the program?  ▢ YES, skip to D5.25 ▢ NO |

code

*Governance and local participation*

| D5.32 | Did you make changes to IMI planning or implementation based on the feedback from your supervisor or someone you supervise?  ▢ YES ▢ NO |
| --- | --- |
| D5.33 | Why or why not? |
| D5.34 | Has **community participation** (feedback/ concerns/ rumors/ resistance) from the community, affected how IMI was implemented in this district?  ▢ YES ▢ NO, skip to D5.36 |
| D5.35 | If yes, how so? |

| D5.28 | Do you have a direct supervisor for your IMI activities?  ▢ YES ▢ NO, skip to D5.31 |
| --- | --- |
| D5.29 | How frequently did your supervisor communicate with you about IMI on a regular basis during IMI rounds?  ▢ Almost Daily ▢ 2-4 times per week  ▢ About once a week ▢ 1-2 times per month  ▢ Less than 1 time per month |
| D5.30 | What actions does your direct supervisor take to supervise your IMI activities? |
| D5.31 | What actions do you take to supervise the IMI activities of those under your supervision? (block) |

code

*Service organization and delivery*

| D5.36 | If IMI were to be implemented again, what would you do differently? |
| --- | --- |
| D5.37 | If you had the freedom to do *anything*, what would you do to improve the efficiency of IMI? |

*Adoption of technology*

| D5.38 | To what extent has your district implemented electronic recording of RCH registers?  ▢ Have not started  ▢ Began implementation (date/time period: )  ▢ Fully implemented (date/time period: ) |
| --- | --- |

*Future priorities*

**(PRESENT TO INTERVIEWEE)**

| D5.57 | To improve your own/district performance, which of the following functions is most important, in your view, to have more freedom about:  *Rank in order of importance; 1 being the most important and 7 being the least important.*  [ ] Increase the amount of the budget you are allowed without higher approval  [ ] Ability to plan and budget with more flexibility [ ] Ability to spend with more flexibility  [ ] Ability to select senior staff [ ] Ability to hire and fire staff  [ ] Ability to set local priorities that override central priorities  [ ] Ability to generate additional local funds  [ ] Other, specify: |
| --- | --- |

code

**Section 5 of 5: Decision capacity**

State: Data collector: District: Date: Block:

*Respondent profile*

*General Overview*

| 1 |  |  |
| --- | --- | --- |
| 2 |  |  |
| 3 |  |  |

| B5.01 | Age (years) : |
| --- | --- |
| B5.02 | Sex : ▢ Male ▢ Female |
| B5.03 | Years in government service : |
| B5.04 | Length of time (*years*) in present block: |
| B5.05 | Current position :  ▢ MO ▢ Other, specify: |
| B5.06 | Were you involved in IMI in any role?  ▢ YES ▢ NO**, END INTERVIEW** |
| B5.07 | Position during IMI :  ▢ MO ▢ Other, specify: _ |
| B5.08 | Were you based in the present block during IMI?  ▢ YES ▢ NO, specify location: |
| B5.09 | Educational qualifications (list highest degree) |
| B5.10 | Additional management training related to your work received in the **past 2 years** (please specify)  Duration (specify  No Title of training days/weeks/months) |

|  | I am going to ask you some questions about the Routine Immunization program (RI) in this Block. |
| --- | --- |
| B5.11 | In this block, has there been progress toward immunization coverage goals in the past 2 years? |
| B5.12 | What are the main challenges of implementing RI in this block?  Encourage free response; note free responses here or use tick boxes if applicable:  ▢ Staffing / Vacancies ▢ Funding  ▢ Inefficiencient operations ▢Vaccine Supply Issues  ▢ Patients lack awareness ▢ Patients hesitancy  ▢ Target population hard to reach ▢ Social media rumors  ▢ Staff motivation ▢ Record-keeping issues |
| B5.13 | How effective has IMI been as a supplement to RI?  ▢ NOT AT ALL EFFECTIVE  ▢ SOMEWHAT EFFECTIVE  ▢ EFFECTIVE  ▢ VERY EFFECTIVE |

code

*Strategic and operational planning*

| B5.16 | *Did you participate in* ***IMI review meetings*** *during IMI rounds?*  ▢ YES ▢ NO, skip to D5.19 |
| --- | --- |
| B5.17 | What is the process for making decisions during IMI review meetings? |
| B5.18 | What changes were made to IMI implementation following the IMI review meetings, if any? |

| B5.14 | What are the **top three** challenges that you have experienced implementing IMI? (List in order of importance)  1.  2.  3. |
| --- | --- |
| B5.15 | What is your role / what specific actions do you take in the development of the final plan of IMI? |

code

*Financial Management*

| B5.24 | In what ways was the training not sufficient? What additional training would have helped you? |
| --- | --- |
| B5.25 | Has your experience with the implementation of IMI led you to make any changes to the RI plan or the way RI is implemented?  ▢ YES ▢ NO, skip to B5.27 |
| B5.26 | If yes, how? |
| D5.27 | Do you think there will be a need for additional rounds of IMI in this block in the future? Why or why not? |

| B5.19 | Were any funds allocated to your block specifically for IMI?  ▢ YES ▢ NO |
| --- | --- |
| B5.20 | Did your block have sufficient funds to implement IMI as planned? ▢ YES ▢ NO |

*Human resources*

| B5.21 | Describe the training that you received to implement IMI. (check all that apply)  ▢ IMI communication session  ▢ BRIDGE IPC Skills training  ▢ AEFI protocol training  ▢ CSO orientation on communication plan and use of monitoring tools  ▢ Media spokespersons training  ▢ IMI microplanning & reporting workshop (district-level)  ▢ Health workers training (ANMs, LHVs, health supervisors)  ▢ Mobilizers training (ASHAs, AWWs)  ▢ Other, specify: |
| --- | --- |
| B5.22 | Did you use this training to solve any problems with IMI planning or implementation in the last year?  ▢ YES ▢ NO |
| B5.23 | Do you feel that this training was sufficient given your responsibilities under the program?  ▢ YES, skip to B5.25 ▢ NO |

| B5.32 | Did you make changes to IMI planning or implementation based on feedback from your supervisor or someone you supervise?  ▢ YES ▢ NO |
| --- | --- |
| B5.33 | Why or why not? |
| B5.34 | Has **community participation** (feedback/ concerns/ rumors/ resistance) from the community, affected how IMI was implemented in this block?  ▢ YES ▢ NO, skip to B5.36 |
| B5.35 | If yes, how so? |

code

*Governance and local participation*

| B5.28 | Do you have a direct supervisor for your IMI activities?  ▢ YES ▢ NO, skip to B5.31 |
| --- | --- |
| B5.29 | How frequently did your supervisor communicate with you about IMI on a regular basis during IMI rounds?  ▢ Almost Daily ▢ 2-4 times per week  ▢ About once a week ▢ 1-2 times per month  ▢ Less than 1 time per month |
| B5.30 | What actions does your direct supervisor take to supervise your IMI activities? |
| B5.31 | What actions do you take to supervise the IMI activities of those under your supervision? (subcentre) |

code

*Service organization and delivery*

| B5.36 | If IMI were to be implemented again, what would you do differently? |
| --- | --- |
| B5.37 | If you had the freedom to do *anything*, what would you do to improve the efficiency of IMI? |

*Adoption of technology*

| B5.38 | To what extent has this block implemented electronic recording of RCH registers?  ▢ Have not started  ▢ Began implementation (date/time period: )  ▢ Fully implemented (date/time period: ) |
| --- | --- |

*Future priorities*

**(PRESENT TO INTERVIEWEE)**

| B5.57 | To improve your own/block performance, which of the following functions is most important, in your view, to have more freedom about:  *Rank in order of importance; 1 being the most important and 7 being the least important.*  [ ] Increase the amount of the budget you are allowed without higher approval  [ ] Ability to plan and budget with more flexibility [ ] Ability to spend with more flexibility  [ ] Ability to select senior staff [ ] Ability to hire and fire staff  [ ] Ability to set local priorities that override central priorities  [ ] Ability to generate additional local funds  [ ] Other, specify: |
| --- | --- |

code

**Section 5 of 5: Decision capacity**

District: Data Collector: Block: Date: Sub-centre:

*Respondent profile General Overview*

| S5.01 | Age : | | | |
| --- | --- | --- | --- | --- |
| S5.02 | Sex : ▢ Male ▢ Female | | | |
| S5.03 | Years in government service : | | | |
| S5.04 | Length of time (*years*) in present sub-centre: | | | |
| S5.05 | Current position :  ▢ ANM ▢ Other, specify: | | | |
| S5.06 | Were you involved in IMI in any role?  ▢ YES ▢ NO**, END INTERVIEW** | | | |
| S5.07 | Position during IMI :  ▢ ANM ▢ Other, specify: | | | |
| S5.08 | Were you based in the present district during IMI?  ▢ YES ▢ NO, specify location: | | | |
| S5.09 | Educational qualifications (list highest degree) | | | |
| S5.10 | Additional management training related to your work received in the **past 2 years** (please specify)  Duration (specify  No Title of training days/weeks/months) | | | |
|  |  | 1 |  |  |
|  |  | 2 |  |  |
|  |  | 3 |  |  |
|  |  | | | |

| S5.11 | *For interviewer: Is other staff present during the interview?*  ▢ YES ▢ NO |
| --- | --- |
| S5.12 | What are the challenges of implementing routine immunization program (RI) in this sub-centre?  Encourage free response; write down responses here and/or use tick boxes if applicable:  ▢ Staffing / Vacancies ▢ Funding  ▢ Inefficiencient operations ▢Vaccine Supply Issues  ▢ Patients lack awareness ▢ Patients hesitancy  ▢ Target population hard to reach ▢ Social media rumors  ▢ Staff motivation ▢ Record-keeping issues |
| S5.13 | How effective has IMI been as a supplement to RI?  ▢ NOT AT ALL EFFECTIVE  ▢ SOMEWHAT EFFECTIVE  ▢ EFFECTIVE  ▢ VERY EFFECTIVE |

code

*Strategic and operational planning Human resources*

| S5.14 | What are the **top three** challenges that you have experienced implementing IMI? (List in order of importance)  1.  2.  3. |
| --- | --- |
| S5.15 | What is your role / what specific actions do you take in the development of the final plan of IMI? |

| S5.16 | Describe the training that you received to implement IMI. (check all that apply)  ▢ IMI communication session  ▢ BRIDGE IPC Skills training  ▢ AEFI protocol training  ▢ CSO orientation on communication plan and use of monitoring tools  ▢ Media spokespersons training  ▢ IMI microplanning & reporting workshop (district-level)  ▢ Health workers training (ANMs, LHVs, health supervisors)  ▢ Mobilizers training (ASHAs, AWWs)  ▢ Other, specify: |
| --- | --- |
| S5.17 | Do you remember what was talked about in the training?  ▢ YES ▢ NO, skip to S5.20 |
| S5.18 | Do you feel that this training was sufficient given your responsibilities under the program?  ▢ YES ▢ NO |
| S5.19 | Why or why not? |


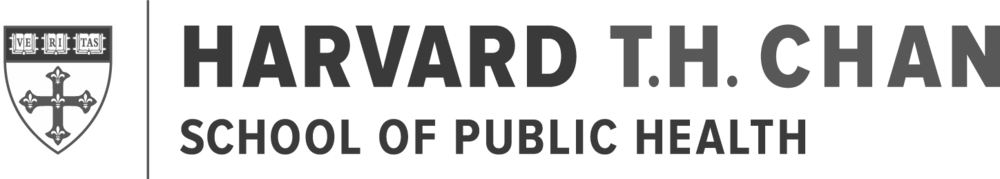

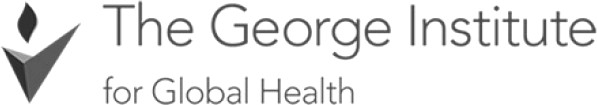


code

| S5.20 | Has your experience with IMI led you to make any changes to the RI plan or the way RI is implemented?  ▢ YES ▢ NO, skip to B5.22 |
| --- | --- |
| S5.21 | If yes, how? |

*Governance and local participation*

| S5.25 | Has **community participation** (feedback/ concerns/ rumors/ resistance) from the community, affected how you implement IMI activities?  ▢ YES ▢ NO, skip to S5.27 |
| --- | --- |
| S5.26 | If yes, how so? |

| S5.22 | Do you have a direct supervisor for your IMI activities?  ▢ YES ▢ NO, skip to S5.25 |
| --- | --- |
| S5.23 | How frequently did your supervisor communicate with you about IMI on a regular basis during IMI rounds?  ▢ Almost Daily ▢ 2-4 times per week  ▢ About once a week ▢ 1-2 times per month  ▢ Less than 1 time per month |
| S5.24 | What actions does your direct supervisor take to supervise your IMI activities? |

*Service organization and delivery*

| S5.27 | If IMI were to be implemented again, what would you do differently? |
| --- | --- |

code

*Availability of infrastructure*

| S5.28 | If you had the freedom to do *anything*, what would you do to improve the efficiency of IMI? |
| --- | --- |

**(OBSERVE, IF POSSIBLE)**

| S5.29 | Subcentre has the following infrastructure (check all that apply) :  ▢ Electricity  ▢ Running water  ▢ Flush or pour toilet  ▢ Pit latrine  ▢ Separate room for ANC check up  ▢ Stand-alone building (i.e. building consists of subcentre only) |
| --- | --- |
| S5.30 | Type of building :  ▢ Government building  ▢ Private building (i.e. house, barn, etc.) |
| S5.31 | Building ownership :  ▢ Owned  ▢ Rented |
| S5.32 | Are toilet facilities usable?  ▢ YES ▢ NO, specify reason:  ▢ No toilet facilities available |

**B Supplementary tables**

Table 1. Results of confirmatory factor analysis for decision space, institutional capacity, and accountability dimensions

| **Indicator** | **Estimate** | **Standard Error** | **Z-score** | ***p*-value** |
| --- | --- | --- | --- | --- |
| *Decision Space* |  |  |  |  |
| Joint decision-making process | 1.00 | - | - | - |
| Added IMI sessions |  |  |  |  |
| *Block* | -0.11 | 0.05 | -2.23 | 0.03 |
| *Subcenter* | 0.19 | 0.05 | 3.82 | 0.00 |
| Added IMI sites | 0.71 | 0.06 | 12.11 | 0.00 |
| Added households to IMI plan |  |  |  |  |
| *Block* | -0.41 | 0.06 | -7.42 | 0.00 |
| *Subcenter* | -0.21 | 0.06 | -3.30 | 0.00 |
| Changes made to IMI based on feedback | -0.27 | 0.06 | -4.81 | 0.00 |
| Local participation | -0.32 | 0.04 | -7.25 | 0.00 |
| *Institutional Capacity* |  |  |  |  |
| Time in current location |  |  |  |  |
| *Block* | 1.00 | - | - | - |
| *Subcenter* | -1.23 | 0.22 | -5.46 | 0.00 |
| Years in government service |  |  |  |  |
| *Block* | 2.20 | 0.30 | 7.35 | 0.00 |
| *Subcenter* | -1.41 | 0.30 | -4.78 | 0.00 |
| Problem solving skills | 0.00 | 0.01 | 0.92 | 0.59 |
| Adoption of technology | 0.01 | 0.02 | 0.92 | 0.36 |
| *Accountability* |  |  |  |  |
| Supervisory actions |  |  |  |  |
| *Block* |  |  |  |  |
| Checked listed households reached | 1.00 | - | - | - |
| Gave feedback | 0.25 | 0.14 | 1.83 | 0.07 |
| Motivated hesitant households | 0.67 | 0.14 | 4.88 | 0.00 |
| *Subcenter* |  |  |  |  |
| Checked listed households reached | -0.41 | 0.13 | -3.29 | 0.00 |
| Visited ANMs in the field | -0.31 | 0.12 | -2.55 | 0.01 |
| Motivated hesitant households | 0.13 | 0.14 | 0.90 | 0.37 |
| Checked logistics | -0.01 | 0.12 | -0.11 | 0.92 |
| Checked supplies | -0.09 | 0.15 | -0.61 | 0.54 |
| Checked microplan | 0.18 | 0.14 | 1.23 | 0.22 |
| Observed service delivery | 0.05 | 0.11 | 0.46 | 0.64 |
| Has a direct supervisor | 0.02 | 0.03 | 0.78 | 0.43 |
| Frequency of communication | 0.03 | 0.03 | 1.34 | 0.18 |
| Community participation | 0.14 | 0.12 | 1.21 | 0.23 |

Note: Indicators with missingness or uniformity causing non-convergence were removed from the CFA.
Final variables used in SEM were those combinations that allowed for model convergence.

Table 2. Validity and reliability measures of latent constructs by model

| **Measure /  Dimension** | **Performance outcome** | | | |
| --- | --- | --- | --- | --- |
|  | *Effectiveness: Proportion reduction in DTP3 coverage gap* | | *Efficiency: Doses delivered per US$1K spent on IMI* | |
|  | Unadjusted | Adjusted | Unadjusted | Adjusted |
| Average variance extracted (AVE) | | |  |  |
| *Decision Space* | **0.61** | **0.64** | **0.52** | **0.51** |
| *Institutional Capacity* | 0.08 | 0.08 | 0.08 | 0.08 |
| *Accountability* | 0.19 | 0.19 | 0.20 | 0.20 |
| Composite reliability |  |  |  |  |
| *Decision Space* | 0.36 | 0.40 | 0.21 | 0.19 |
| *Institutional Capacity* | 0.00 | 0.00 | 0.00 | 0.00 |
| *Accountability* | 0.32 | 0.32 | 0.33 | 0.32 |

**References**

World Bank Group, 2010. Decentralization of health in the Indian state of West Bengal : analysis of decision space, institutional capacities and accountability.
